# EGR1 and RXRA transcription factors link TGF-β pathway and CCL2 expression in triple negative breast cancer cells

**DOI:** 10.1038/s41598-021-93561-6

**Published:** 2021-07-08

**Authors:** Alisa M. Gorbacheva, Aksinya N. Uvarova, Alina S. Ustiugova, Arindam Bhattacharyya, Kirill V. Korneev, Dmitry V. Kuprash, Nikita A. Mitkin

**Affiliations:** 1 Center for Precision Genome Editing and Genetic Technologies for Biomedicine, Engelhardt Institute of Molecular Biology, Russian Academy of Sciences, Moscow, 119991 Russia; 2 Immunology Laboratory, Department of Zoology, University of Calcutta, 35, Ballygunge Circular Road, Kolkata, West Bengal 700019 India; 3 Biological Faculty, Lomonosov Moscow State University, Moscow, 119234 Russia

**Keywords:** Chemokines, Transcriptional regulatory elements, Immunogenetics

## Abstract

Transforming growth factor beta (TGF-β) is the main cytokine responsible for the induction of the epithelial-mesenchymal transition of breast cancer cells, which is a hallmark of tumor transformation to the metastatic phenotype. Recently, research demonstrated that the chemokine CCL2 gene expression level directly correlates with the TGF-β activity in breast cancer patients. CCL2 attracts tumor-associated macrophages and is, therefore, considered as an important inductor of breast cancer progression; however, the precise mechanisms underlying its regulation by TGF-β are unknown. Here, we studied the behavior of the CCL2 gene in MDA-MB-231 and HCC1937 breast cancer cells representing mesenchymal-like phenotype activated by TGF-β. Using bioinformatics, deletion screening and point mutagenesis, we identified binding sites in the CCL2 promoter and candidate transcription factors responsible for its regulation by TGF-β. Among these factors, only the knock-down of EGR1 and RXRA made CCL2 promoter activity independent of TGF-β. These factors also demonstrated binding to the CCL2 promoter in a TGF-β-dependent manner in a chromatin immunoprecipitation assay, and point mutations in the EGR1 and RXRA binding sites totally abolished the effect of TGF-β. Our results highlight the key role of EGR1 and RXRA transcription factors in the regulation of CCL2 gene in response to TGF-β pathway.

## Introduction

Transforming growth factor beta (TGF-β) is a cytokine that acts as a tumor suppressor in normal conditions and in the early stages of breast cancer (BC) development^1^. BC progression leads to the modification of TGF-β-dependent signaling pathways, which results in the altered response of a cancer cells to TGF-β, the elevated production of TGF-β, and modification of the tumor microenvironment^2^. In particular, enhanced TGF-β production is associated with the differentiation of myeloid-derived suppressor cells and regulatory T-helpers, which provoke tumor development by the inhibition of local inflammatory processes^3^. One of the key pathogenic functions of TGF-β is the induction of the epithelial-mesenchymal transition that results in the loss of the epithelial phenotype of the tumor and its transition to the aggressive metastatic stage^4^. This process is accompanied by activation of the range of genes responsible for the invasion and migration of cancer cells. A series of studies demonstrated that TGF-β was able to activate the expression of chemokines that enhance the metastatic potential of tumor cells^5^. Specifically, TGF-β induced the expression levels of CXCL1—attracts myeloid cells in the primary tumor site^6^, CXCL5—attracts neutrophils^7^, and CCL20—stimulates proliferative and migrative activity of BC cells^8^.

However, until recently, there was no information regarding the effect of TGF-β on CCL2 chemokine expression in BC cells, and the association of increased TGF-β/Smad3 expression with CCL2 induction in smooth muscle cells is poorly translated to cancer cells^9^. CCL2 (classically named MCP-1, monocyte chemoattractant protein 1), is known to be a powerful monocyte chemoattractant and to strongly induce metastasis formation by BC cells. In particular, lung metastases are associated with the involvement of inflammatory monocytes expressing the CCR2 receptor, which differentiate into metastasis-associated macrophages that prepare the microenvironment for cancer cells extravasation, as well as stimulate tumor growth at the site of metastasis^10^. CCL2 has also been shown to play a key role in attracting tumor-associated macrophages (TAM) to the primary tumor site^11^. TAM contributes to tumor progression by inducing angiogenesis, intensifying the epithelial–mesenchymal transition, and stimulating the intravasation of cancer cells^12^. In general, the elevated expression of CCL2 by BC cells is associated with an aggressively metastasizing tumor phenotype and with poor prognoses in patients^13^.

Recent studies demonstrated that the CCL2 gene expression level directly correlates with the TGF-β activity in breast cancer patients. These data are supported by the fact that, in the 4T1-BALB/c BC allograft mouse model, the inhibition of TGF-β causes a significant reduction in the levels of CCL2 expression in the primary tumor site, which correlates with lower levels of lung metastases^14^. In addition, it has been reported that TGF-β effects on CCL2 expression strongly depend on BC cell phenotype and activation is typical for advanced-stage BC cells^14^. In the current study, we aimed to identify the mechanism underlying regulation of CCL2 gene expression by TGF-β in advanced-stage triple-negative BC cells with mesenchymal-like phenotype.

## Results

### Characterization of potential CCL2 promoter region

**Figure 1:**
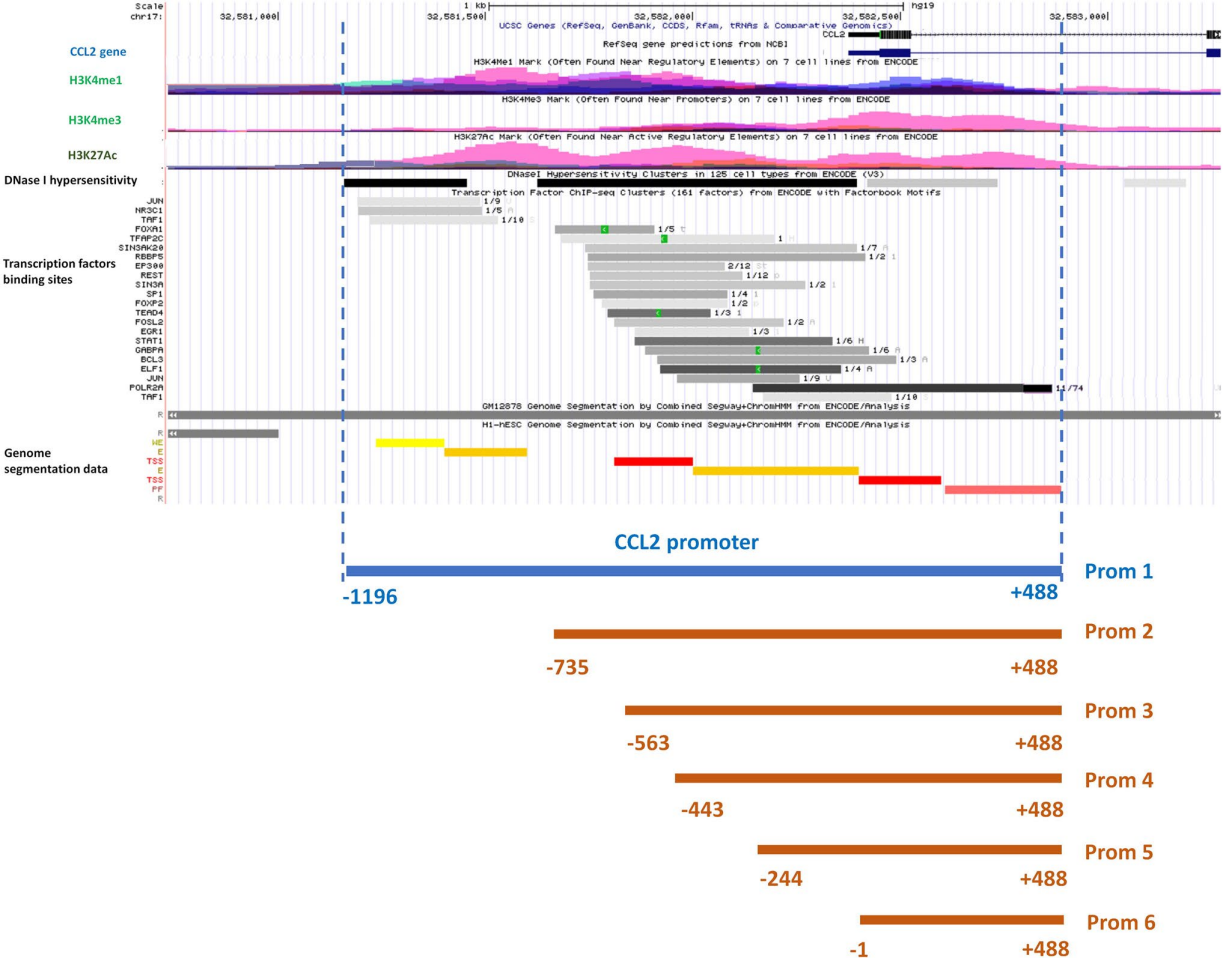
Illustration of the 5′ area of the human CCL2 gene (UCSC Genome Browser, Human Feb. 2009 GRCh37/hg19 Assembly) with mapped potential CCL2 promoter variants used for deletion screening. The numbers indicate positions in response to the CCL2 transcription start site. *H3K4Me1* the track indicating areas of monomethylation of Histone H3 at lysine 4, *H3K4Me3* the track indicating areas of trimethylation of Histone H3 at lysine 4, *H3K27Ac* the track indicating areas of acetylation of Histone H3 at lysine 27, *DNase I hypersensitivity* the track indicating deoxyribonuclease I hypersensitivity clusters.

The CCL2 promoter region was not described and characterized earlier; therefore, we performed its identification using the following epigenetic data represented in the UCSC Genome Browser (http://genome.ucsc.edu/, Human Feb. 2009 GRCh37/hg19 Assembly) as described earlier^15^: DNase-I hypersensitivity clusters, histone modifications (H3K4me3, H3K4me1, and H3K27ac), the localization of transcription factor (TF) binding sites, and information on chromatin segmentation that combines data on different epigenetic parameters^16^. Based on these data, we identified the region spanning from − 1196 to + 488 bp from the CCL2 transcription start site (TSS) as the potential promoter (Fig. 1).

### TGF-β1 induces CCL2 gene expression and CCL2 promoter activity in advanced stage triple-negative breast cancer cell lines

**Figure 2:**
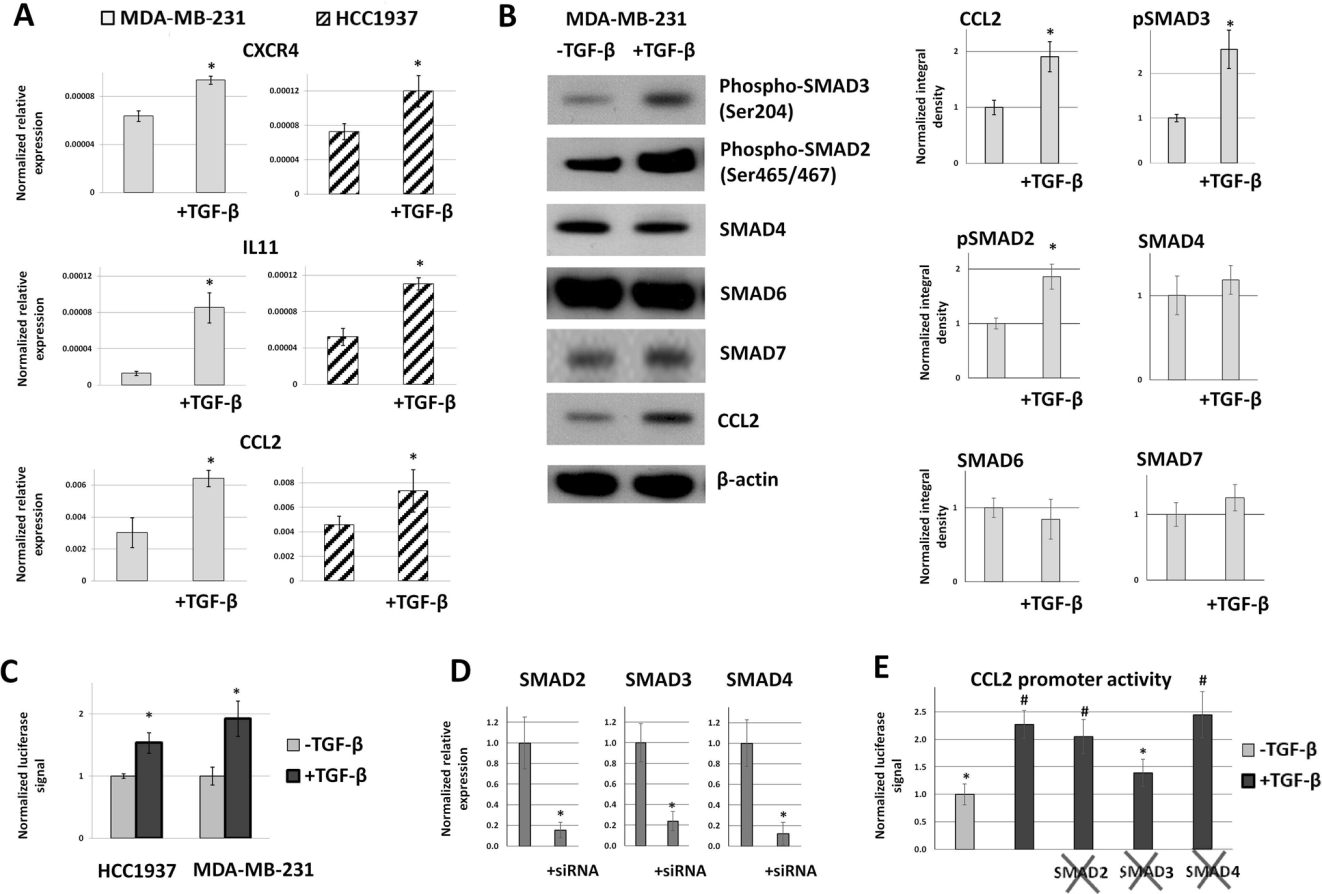
Transforming growth factor beta (TGF-β1)-induced activation of advanced stage triple-negative breast cancer cell lines enhanced CCL2 gene expression and CCL2 promoter activity. (**A**) The incubation of MDA-MB-231 and HCC1937 cells with 10 ng/mL TGF-β1 for 24 h led to significant induction of the expression levels of IL-11 and CXCR4 genes (the main TGF-β-induced targets) and also resulted in elevated CCL2 mRNA levels. mRNA levels were measured by RT-PCR in real time. The results of five independent experiments are represented. *P < 0.01. (**B**) The incubation of MDA-MB-231 cells with TGF-β1 led to the induction of phospho-SMAD3 (Ser 204), phospho-SMAD2 (Ser465/467) and CCL2 protein levels with no influence on SMAD4, SMAD6 and SMAD7 protein levels. Representative western blots are shown (left panel). For chemiluminescent detection of the bands corresponding to SMAD, CCL2 and β-actin proteins we applied X-ray film exposure. The full-length blot images are represented in Supplementary Fig. S1. The experiment was repeated three times, the images were quantified using ImageJ software (right panel). The values of integral densities for CCL2, pSMAD3, pSMAD2, SMAD4, SMAD6 and SMAD7 are normalized on the respective β-actin bands. Normalized integral densities for CCL2, pSMAD3, pSMAD2, SMAD4, SMAD6 and SMAD7 in MDA-MB-231 cells without TGF-β1 activation are represented as 1. *P < 0.01. (**C**) TGF-β induced CCL2 promoter activity in a luciferase reporter system in both MDA-MB-231 and HCC1937 cell lines. The data were obtained in five independent experiments and normalized to the Renilla luciferase activity. Normalized luciferase signals in MDA-MB-231 and HCC1937 cells without TGF-β1 activation are represented as 1. *P < 0.01. (**D**) The efficiency of knock-down of SMAD2, SMAD3 and SMAD4 genes in MDA-MB-231 cells. For each gene, real-time PCR signal was normalized to the sample without siRNA. The data are represented as the mean ± SEM (five independent experiments). *P < 0.01 in respect to control without siRNA. (**E**) TGF-β induced CCL2 promoter activity in the presence of SMAD2-, SMAD3 or SMAD4-specific siRNAs in a luciferase reporter system in MDA-MB-231 cells (data from five independent experiments). Luciferase signal without TGF-β1 normalized to Renilla luciferase activity is represented as 1. *P < 0.01 in respect to control with TGF-β1; ^#^P < 0.01 in respect to control without TGF-β1.

In the current study, we used MDA-MB-231 and HCC1937 triple negative human breast cancer cell lines that are reported to overexpress CCL2^17^ and demonstrate mesenchymal-like phenotype^18,19^. We verified that TGF-β could induce CCL2 expression in triple-negative breast cancer cells with mesenchymal-like phenotype as it was indicated in previously published data^14^. The incubation of MDA-MB-231 and HCC1937 cells with 10 ng/mL of recombinant TGF-β1 protein (ab50036, Abcam, Cambridge, UK) for 24 h led to significant induction of expression levels of the IL-11 and CXCR4 genes (Fig. 2A), which are considered as the main TGF-β-induced targets^1^. The incubation of MDA-MB-231 cells with TGF-β1 resulted in higher levels of the phospho-SMAD3 (Ser 204) and phospho-SMAD2 (Ser465/467) forms, the key participants of canonical TGF-β intracellular cascade^20^. All of these TGF-β-dependent effects correlated with the stimulation of both CCL2 mRNA (Fig. 2A) and protein (Fig. 2B) levels that indicated the response of CCL2 on TGF-β stimulation.

In addition, incubation of MDA-MB-231 and HCC1937 breast cancer cells with TGF-β1 (10 ng/mL, for 24 h) led to a 1.5–twofold increase in the level of CCL2 promoter activity in the luciferase reporter system (Fig. 2C). These results highly correlate with the expression data and confirm the role of TGF-β signaling in the regulation of the CCL2 promoter.

To perform a more comprehensive analysis of SMAD proteins in TGF-β1-dependent regulation of CCL2 promoter we measured the levels of several other SMAD family members, SMAD4, SMAD6 and SMAD7 (Fig. 2B). SMAD4 is known to form a complex with activated p-SMAD2/3 which acts as a direct transcriptional activator of TGF-β-dependent target genes^21,22^. We detected no change in SMAD4 protein expression in response to TGF-β1 while the levels of p-SMAD2 and p-SMAD3 were induced. Apparently, SMAD4 activation is not required for the complex formation because the protein is already present in the cytoplasm and is recruited to the complex in a non-phosphorylated form^21^. Efficient complex formation is supported by the induction of IL11 and CXCR4 that have been reported to be direct p-SMAD2/3-SMAD4 target genes^23^. SMAD6 and SMAD7 have been shown to reduce the activity of p-SMAD2/3-SMAD4 complex acting in an antagonistic way^22^ and are therefore considered as inhibitory members of the SMAD family. The unchanged SMAD6 and SMAD7 proteins levels support the activating nature of intracellular TGF-β receptor signaling in MDA-MB-231 cells.

In order to check whether p-SMAD2/3-SMAD4 complex is able directly activate the CCL2 gene, we tested CCL2 promoter activity in TGF-β-induced MDA-MB-231 cells in the presence of specific siRNAs against SMAD2, SMAD3 and SMAD4 (Fig. 4D,E). Only SMAD3 knockdown significantly abrogated CCL2 promoter activation after TGF-β treatment indicating that other proteins of SMAD family do not interact directly with the promoter.

### − 244/− 1 region of the CCL2 promoter is responsible for its TGF-β-dependent regulation

**Figure 3:**
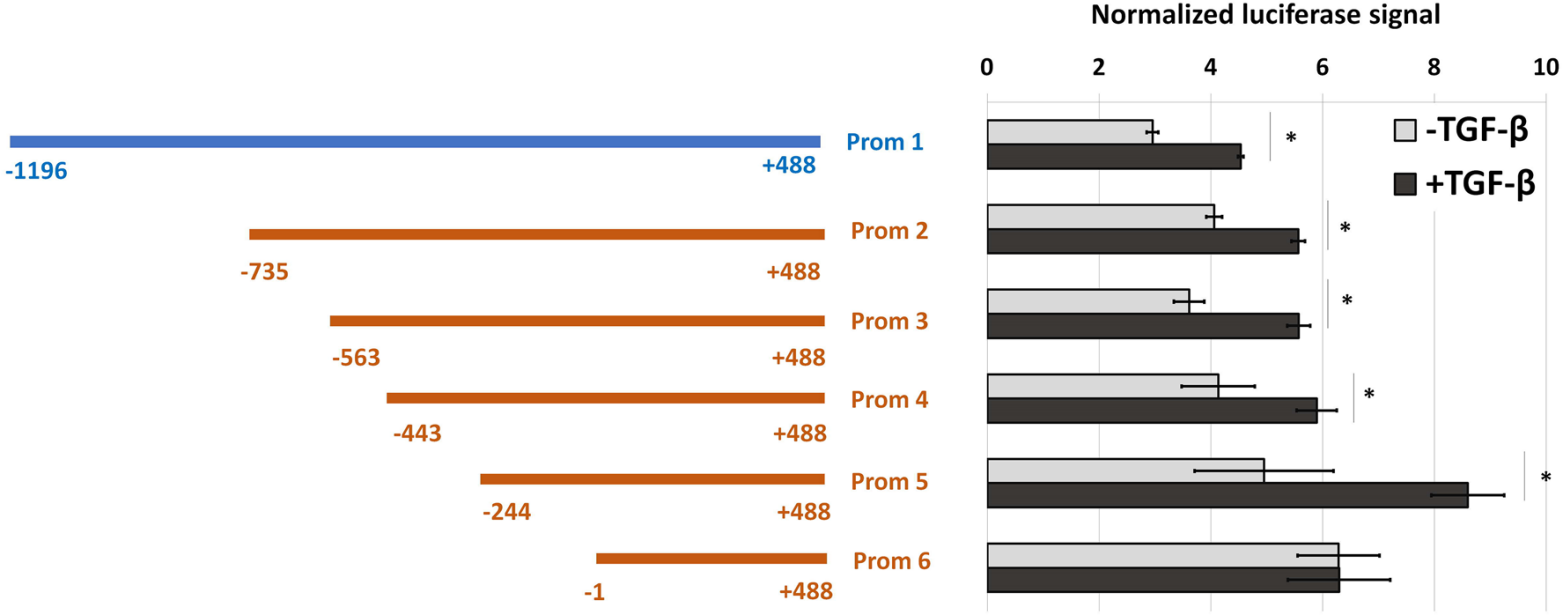
The − 244/− 1 region of the CCL2 promoter is responsible for its TGF-β1-dependent regulation. Functional analysis of deletion variants of the CCL2 promoter. Left, schematic illustration of promoter variants with positions in response to the CCL2 transcription start site. Right, normalized luciferase signals in MDA-MB-231 breast cancer cells in normal conditions and after the incubation with TGF-β1 (10 ng/mL, 24 h). Note that shorter promoter fragments yield smaller plasmid sizes and better transfection efficiencies, resulting in a gradual increase of the overall luciferase signal. The data were obtained in five independent experiments and normalized to the Renilla luciferase activity. *P < 0.01.

We applied deletion screening of CCL2 promoter to identify its region responsible for TGF-β1-dependent activation. The activities of luciferase reporter constructs containing deletion variants of the CCL2 promoter were assessed in MDA-MB-231 breast cancer cells in the presence or absence of TGF-β1 (10 ng/mL, 24 h), (Fig. 3). All promoter regions except Prom 6 demonstrated the dependence on TGF-β-induced activation. In the case of Prom 6, this effect was completely absent, although this region is essential for maintaining basal promoter activity. Comparing the character of influence of TGF-β1 on the activities of Prom 5 and Prom 6, the region is likely located within positions − 244 and − 1 in response to CCL2 transcription start site should be responsible for the dependence of CCL2 promoter on TGF-β.

### EGR1 and RXRA transcription factors are essential for TGF-β-dependent activation of the CCL2 promoter

To identify potential transcription factors binding sites within the − 244/− 1 region of the CCL2 promoter we used the MoLoTool (http://molotool.autosome.ru) online service, which allows the search for transcription factor binding sites in a given sequence using curated models of these sites presented in the HOCOMOCO database (http://hocomoco11.autosome.ru/)^24^. For the first step, we formed a list of transcription factors with “strong” (− log10(P-value) > 5) predicted sites within the 244/− 1 region: MAZ, SP4, EGR2, THAP1, VEZF1, PAX6, SP1, RXRA, WT1, SP3, ZN768, BCL6, ZSC22, SP2, KLF3, RARA, ZBT14, and EGR1. In the next step, based on published data, we selected those factors from the list that are known to be involved in TGF-β-induced cascades: EGR1^25^, EGR2^26^, PAX6^27^, RARA and RXRA^28^, and SP1^29^. All selected transcription factors demonstrated high expression levels in both MDA-MB-231 and HCC1937 cell lines according to the mRNA expression data represented in the Broad Institute Cancer Cell Line Encyclopedia (Supplementary Table S2), which was used as an additional selection criterion.

**Figure 4:**
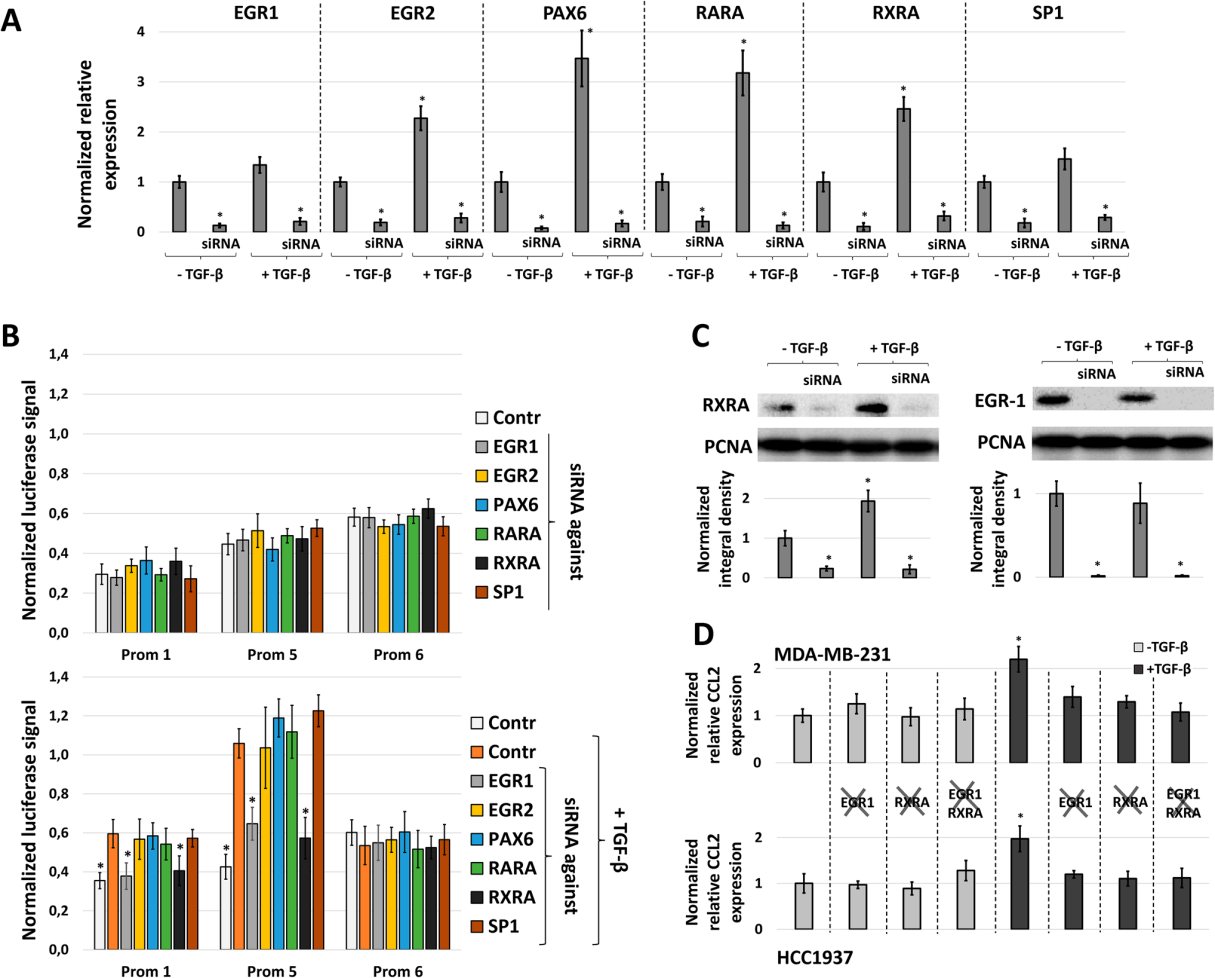
Knock-down of EGR1 and RXRA transcription factors resulted in the independence of the CCL2 promoter of TGF-β1 activation in MDA-MB-231 breast cancer cells. (**A**) The efficiency of knock-down of EGR1, EGR2, PAX6, RARA, RXRA, and SP1 genes after transfection of MDA-MB-231 cells with specific siRNAs in the presence or absence of TGF-β1 activation was tested by real-time PCR. For each gene, the normalization to the sample without siRNA and TGF-β1 was performed (the meanings for these samples are represented as 1). The real-time PCR data are represented as the mean ± SEM (five independent experiments). *P < 0.01 in respect to control without siRNAs and TGF-β1. (**B**) Prom 1, Prom 5, and Prom 6 CCL2 promoter variants were tested using a luciferase reporter assay in MDA-MB-231 cells transfected with specific siRNAs in normal conditions (top panel) or in the presence of TGF-β1 (10 ng/mL, 24 h) (bottom panel). The data were obtained in seven independent experiments and normalized to the Renilla luciferase activity. *P < 0.01 in respect to control with TGF-β1. (**C**) EGR1 and RXRA protein levels measured in nuclear extracts of MDA-MB-231 cells in the presence or in the absence of specific siRNAs and/or TGF-β1 (10 ng/mL, 24 h). The experiment was repeated for three times. Representative western blots are shown (upper panel). For the detection of RXRA, EGR1 and PCNA proteins the membranes were splitted horizontally into two pieces. The upper piece of each membrane was stained with anti-RXRA or anti-EGR1 primary antibodies, and the lower—with anti-PCNA antibodies. Chemiluminescent detection of the bands was performed using ChemiDoc Imager (Bio-Rad Laboratories, Hercules, CA, USA). Full-size images of splitted membranes are represented in Supplementary Fig. S2. Membrane images were quantified using ImageJ software (bottom panel). Integral densities for EGR1 and RXRA bands normalized to PCNA in MDA-MB-231 cells without TGF-β1 activation are represented as 1. *P < 0.01. (**D**) CCL2 mRNA levels were estimated by RT PCR in real time. For each cell line, samples without siRNAs and TGF-β1 are represented as 1. The results of three independent experiments are shown. The full-length blot images are represented in Supplementary Fig. S2. *P < 0.01 as compared to control without siRNAs and TGF-β1.

To reveal the roles of these factors in CCL2 promoter regulation, we estimated the activity of the full-size CCL2 promoter (Prom 1) and its Prom 5 and Prom 6 fragments in MDA-MB-231 cells in normal conditions and in the presence of TGF-β1 (10 ng/mL, 24 h) or/and specific siRNAs against EGR1, EGR2, PAX6, RARA, RXRA, and SP1 factors mRNAs. The efficiency of siRNAs in suppressing the expression of these factors was preliminarily estimated by qPCR in the MDA-MB-231 cell line and was not less than 80% (Fig. 4A). TGF-β1 induced expression of all of the studied transcription factors except for EGR1 and SP1^30,31^. siRNA knock-downs of all the factors abolished the activation of the corresponding genes and in the absence of TGF-β1 had no effects on the activity of CCL2 promoter variants (Fig. 4B, top panel). The Prom 6 fragment demonstrated no dependence on both TGF-β1 activation and the levels of any of the studied factors (Fig. 4B, bottom panel). The knock-down of any of EGR1 or RXRA completely abolished the effect of Prom1 and Prom 5 stimulation in response to TGF-β1. According to these results, EGR1 and RXRA could be considered as the main mediators of TGF-β1-dependent regulation of the CCL2 promoter.

Transfection of MDA-MB-231 cells with EGR1- or RXRA-specific siRNAs led to reduced levels of the respective proteins in the nucleus (Fig. 4C) and these levels could not be induced by TGF-β1 (10 ng/mL, 24 h). In the absence of specific siRNAs, TGF-β1 treatment resulted in elevated RXRA protein levels in the nucleus and did not influence EGR1 protein level, in agreement with mRNA expression data (Fig. 4A). At the level of the CCL2 mRNA, either EGR1- or RXRA-specific siRNA abrogated the TGF-β1-triggered effect almost as completely as both siRNAs together, both in MDA-MB-231 and in HCC1937 cells (Fig. 4D).

### Both EGR1 and RXRA bind to the CCL2 promoter after TGF-β treatment independently in a time-dependent manner

**Figure 5:**
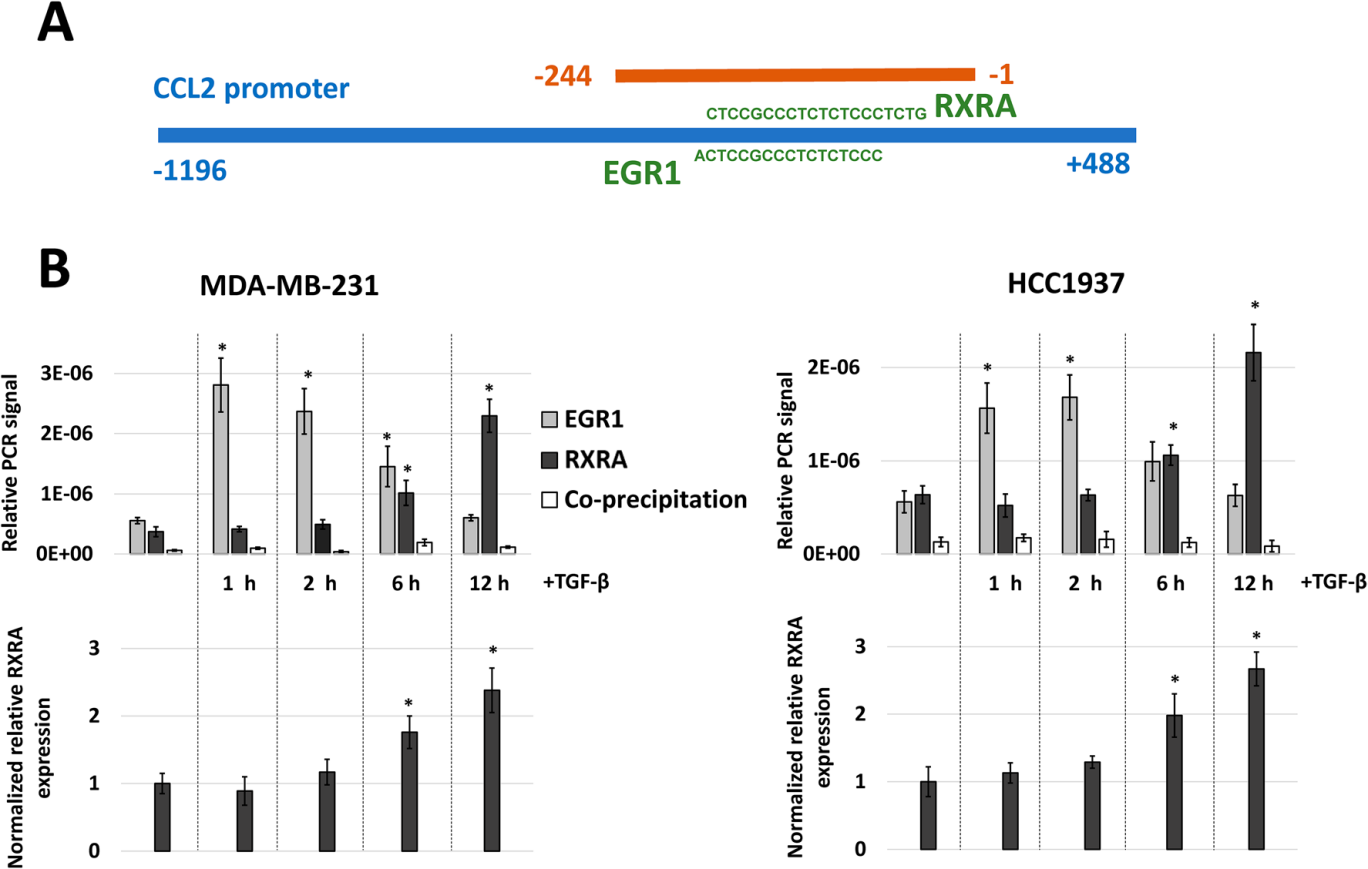
TGF-β1 induces EGR1 and RXRA binding to the CCL2 promoter in MDA-MB-231 and HCC1937 breast cancer cells in time-dependent manner. (**A**) Map of the CCL2 promoter, predicted EGR1-/RXRA-binding sites, and PCR product amplified in the chromatin immunoprecipitation (ChIP) assay. (**B**) Activation of MDA-MB-231 and HCC-1937 cells with TGF-β1 led to elevated time-dependent EGR1 and RXRA crosslinking to the amplicon containing corresponding consensus sites (top panel). As described Material and Methods, none of the specificity controls produced signals above background. TGF-β1 also induced RXRA expression as estimated by RT-PCR in real time (bottom panel). The RT-PCR data were normalized to the sample without TGF-β1 separately for each cell line. The results of three independent experiments are represented both for CHIP-assay and RT-PCR. *P < 0.01 in response to control without TGF-β1.

To estimate the influence of TGF-β on the efficiency of EGR1 and RXRA binding to the CCL2 promoter in vivo, we used a classical chromatin immunoprecipitation (ChIP) assay. As the EGR1- and RXRA-binding sites are located in the same (− 244/− 1) region of the promoter, we analyzed the amount of cross-linked DNA from MDA-MB-231 and HCC1937 cells precipitated with anti-EGR1 and anti-RXRA antibodies using the primers corresponding to this region (schematically illustrated in Fig. 5A). We performed ChIP analysis for both cell lines activated by TGF-β1 (10 ng/mL) for 1, 2, 6 and 12 h. In order to determine whether RXRA and EGR1 could cooperatively bind to the same region of the promoter we also performed co-immunoprecipitation i.e. after precipitation with anti-EGR1 antibodies an additional precipitation round was carried out with anti-RXRA antibodies. We also controlled RXRA expression level as this factor is induced by TGF-β1, unlike EGR1 mRNA and protein (Fig. 4A). EGR1 demonstrated maximal binding to CCL2 promoter already at 1 h of TGF-β1 treatment (Fig. 5B), in agreement with existing data on direct EGR1 activation by TGF-β1^30^. The strength of RXRA binding to CCL2 promoter also correlated with its expression level (Fig. 5B). These data indicate time-resolved binding of EGR1 and RXRA transcription factors to CCL2 promoter in response to TGF-β, suggesting largely independent action of these trascription on CCL2 gene expression. Indeed, crosslinked chromatin fragments precipitated with anti-EGR1 antibodies could not be precipitated with anti-RXRA antibodies in co-immunoprecipitation assay. The strength of combined EGR1 and RXRA binding to CCL2 promoter correlates well with TGF-β1-induced CCL2 expression and promoter activity (Fig. 2) and provides a mechanistic explanation for transcriptional activation of the CCL2 gene by these two factors for an extended period of time.

### Point mutations in EGR1 and RXRA binding sites in CCL2 promoter completely abolished its activation by TGF-β

**Figure 6:**
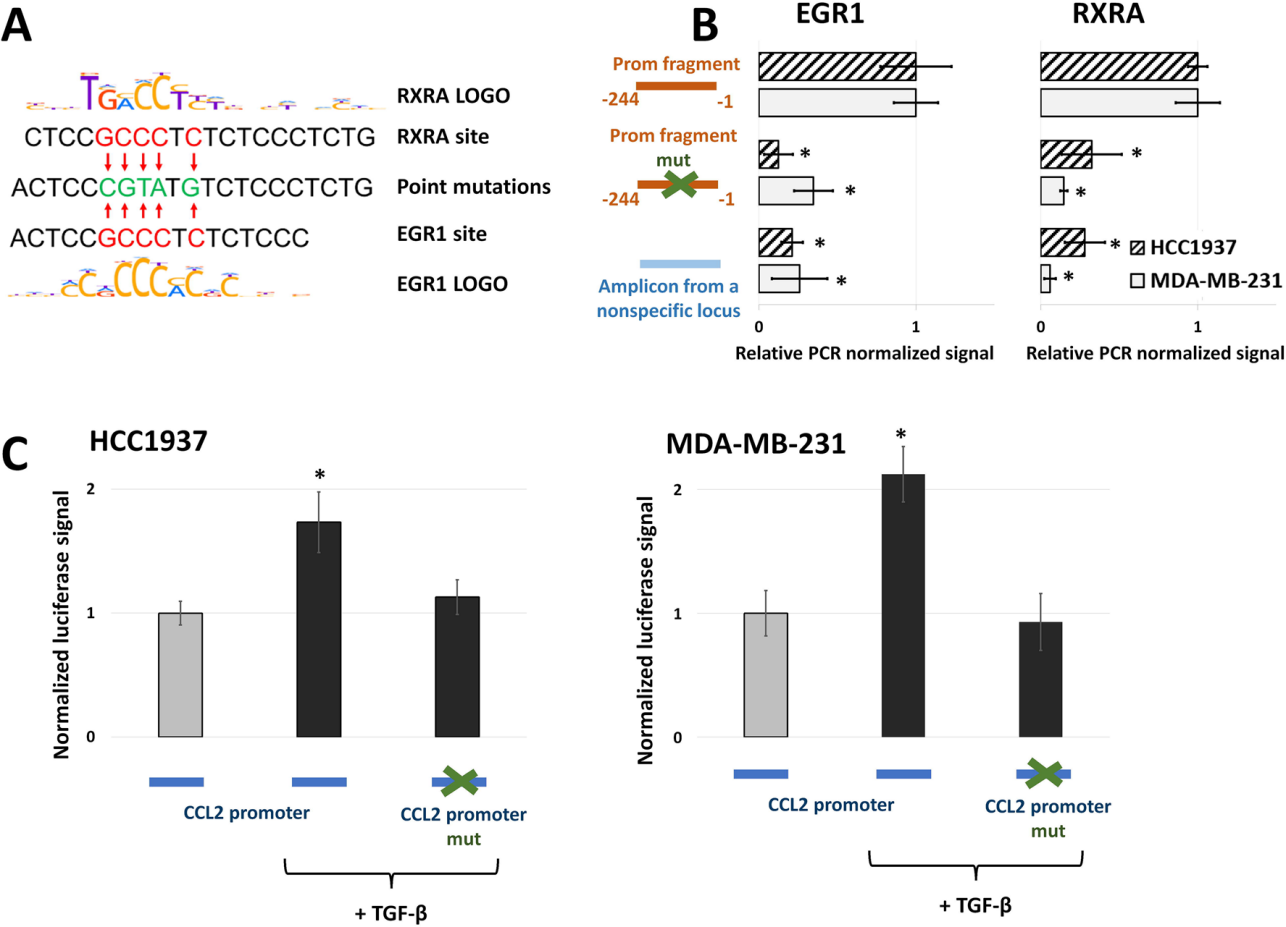
Point mutations in the EGR1 and RXRA binding sites in the CCL2 promoter completely abolished its activation by TGF-β1. (**A**) Position weight matrixes of the EGR1 and RXRA binding sites and the strategy of their point mutagenesis. Red arrows indicate nucleotides changes. (**B**) Point mutations of the EGR1/RXRA1 sites in the CCL2 promoter totally abrogated EGR1 and RXRA1 binding to the promoter. The efficiency of EGR1 and RXRA1 binding to different variants of the CCL2 promoter was estimated by pull-down assay with the use of nuclear extracts from MDA-MB-231 and HCC1937 breast cancer cells. The data were normalized to the normal − 244/− 1 amplicon after subtraction of the background signal (the signal for this sample is represented as 1 for each cell line). The results of five independent experiments are represented. *P < 0.01. (**C**) Mutation of the EGR1/RXRA binding sites resulted in a full absence of the effect of TGF-β1 (10 ng/mL, 24 h) on CCL2 promoter activity in both the MDA-MB-231 and HCC1937 cell lines as estimated using a luciferase reporter assay. The data were obtained in five independent experiments and normalized to the Renilla luciferase activity. Normalized luciferase signals of original CCL2 promoter without TGF-β1 activation are represented as 1 for each cell line. *P < 0.01.

In order to verify the roles of the EGR1 and RXRA transcription factors in TGF-β-dependent regulation of the CCL2 promoter, we generated a luciferase reporter construct containing the CCL2 promoter with point mutations in the EGR1 and RXRA sites. The EGR1- and RXRA-binding sites in CCL2 promoter were overlapped, and their consensus sequences are highly similar; therefore, mutations in their key positions should damage both of them (schematically illustrated on the Fig. 6A).

Preliminary, we applied a pull-down assay^32^ to test the influence of these mutations on the efficiency of EGR1 and RXRA binding to the CCL2 promoter. For this reason, we generated two types of amplicons: the − 244/− 1 region of the CCL2 promoter and the same region with point mutations in the EGR1/RXRA sites (Fig. 6B). For precipitation, we used nuclear extracts from MDA-MB-231 and HCC1937 cells and anti-EGR1 or anti-RXRA antibodies. We concluded that the introduced mutations totally abrogated EGR1 and RXRA1 binding to the CCL2 promoter in both cell lines (Fig. 6B).

In the next step, we compared the activity of a full-size CCL2 promoter and its mutated variant in normal conditions and in the presence of TGF-β1 (10 ng/mL, 24 h) (Fig. 6C). Mutation of the EGR1/RXRA binding sites resulted in a full absence of the effect of TGF-β1 on CCL2 promoter activity in both the MDA-MB-231 and HCC1937 cell lines. This result agrees with both the pull-down and CHIP data and indicates the exclusive role of the EGR1/RXRA binding sites in the TGF-β-dependent regulation of the CCL2 promoter.

## Discussion

A direct correlation between the CCL2 and TGF-β levels is typical for progressive stages of breast cancer as was recently shown using both clinical samples and in vitro studies^14^. In vivo studies applying the 4T1-BALB/c BC mouse model demonstrated that the inhibition of TGF-β causes a significant reduction in the levels of CCL2 expression in the primary tumor site, which correlates with lower levels of lung metastases^14^. We verified these data using MDA-MB-231 and HCC1937 triple negative human breast cancer cell lines, which are reported to overexpress CCL2^17^. These cells demonstrated a response to TGF-β1 that was detected by the induction of the expression levels of the main TGF-β targets^1^, IL-11 and CXCR4 (Fig. 2A), and higher levels of phospho-SMAD3 (Ser 204) and phospho-SMAD2 (Ser465/467) (Fig. 2B), which are considered as the key participant of the TGF-β intracellular cascade^20^. All of these TGF-β-dependent effects correlated with stimulation of the CCL2 mRNA (Fig. 2A) and protein (Fig. 2B) levels that confirm the previously published data. Thereby, TGF-β undoubtedly acts as an activator of CCL2 expression in progressive breast cancer; however, the molecular mechanism of this action remains unknown.

Using the current epigenetic data presented in the UCSC Genome Browser, we identified the region spanning from − 1196 to + 488 from the CCL2 transcription start site as a potential CCL2 promoter. TGF-β1-induced activation of MDA-MB-231 and HCC1937 breast cancer cells led to an increase in the activity of the CCL2 promoter in the luciferase reporter system (Fig. 2C), and this was fully provided by its − 244/− 1 region (Fig. 3). It should be noted that p-SMAD2/3-SMAD4 complex, which is considered as the canonical effector of intracellular signaling of TGF-β receptor acting as a transcriptional activator^21,22^, is not involved in direct CCL2 promoter activation since only SMAD3 knockdown but not that of SMAD2 or SMAD4 influenced the level of CCL2 promoter activity after TGF-β1 treatment (Fig. 2D,E). Bioinformatical analysis of 244/− 1 TGF-β-dependent region made it possible to predict potential binding sites for the transcription factors. The extensive list of transcription factors has been shortened as a result of literature annotation. We were interested only in the factors whose activity is described as dependent on TGF-β-signaling in cancer cells: EGR1^25^, EGR2^26^, PAX6^27^, RARA and RXRA^28^, and SP1^29^. Among these factors, the knock-down of only EGR1 and RXRA completely abolished the effect of CCL2 promoter activation in response to TGF-β1 (Fig. 4B). Interestingly, RARA knock-down did not have an effect on the promoter activity, indicating that RXRA probably binds CCL2 promoter as a homodimer. Additional experiments supported the role of EGR1 and RXRA in TGF-β-dependent regulation of the CCL2 promoter as their knock-down resulted in the absence of TGF-β-induced increase in CCL2 expression (Fig. 4D), both factors demonstrated binding to the promoter in a TGF-β1-dependent manner (Fig. 5), and point mutagenesis of their common binding site led to the loss of the ability of TGF-β1 to activate the CCL2 promoter (Fig. 6). Of note, EGR1 and RXRA bind to the CCL2 promoter independently but with different kinetics which may explain their essential and independent roles in TGF-β-induced CCL2 promoter activation.

**Figure 7:**
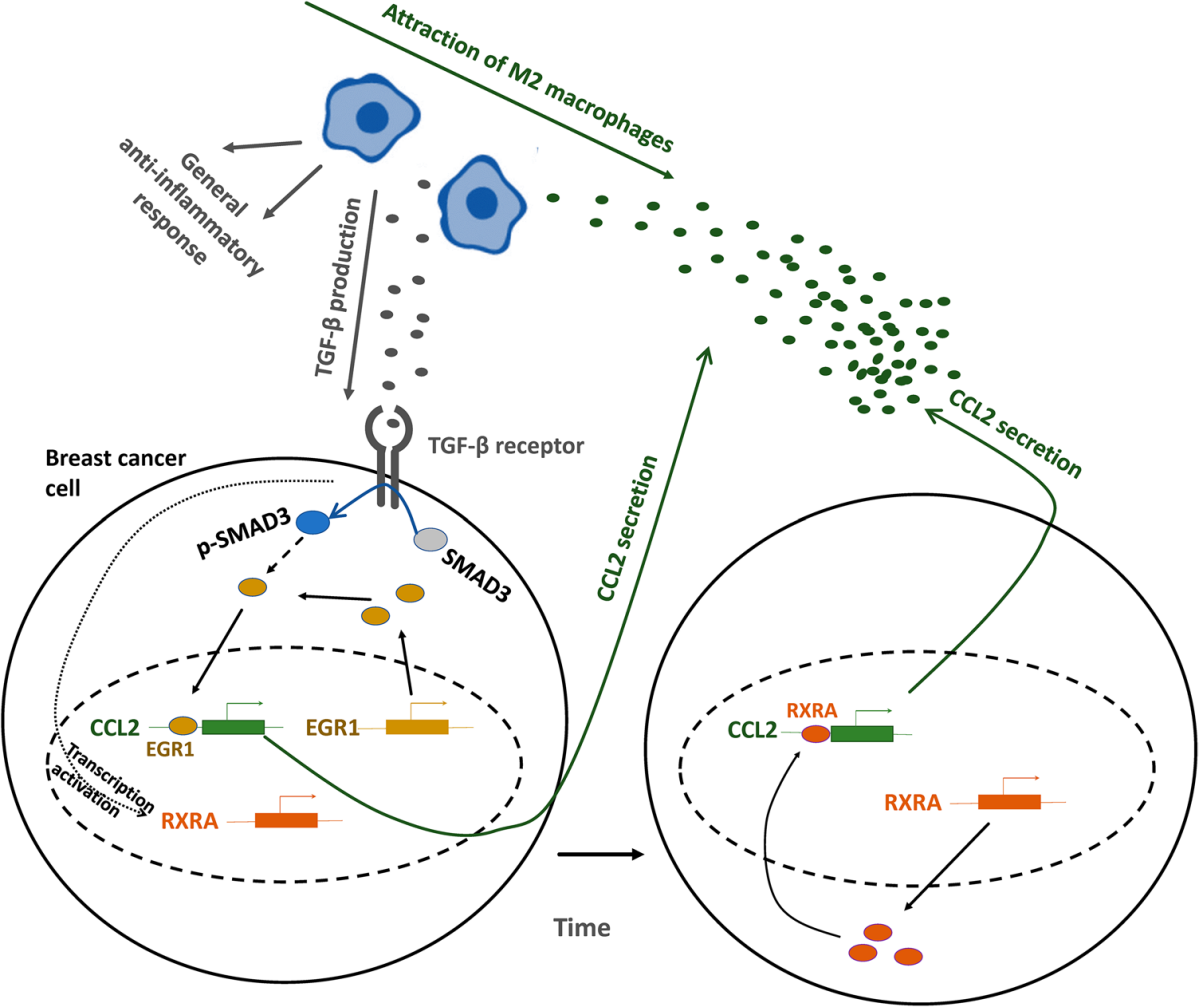
Schematic illustration of potential EGR1 and RXRA participation in TGF-β-CCL2 positive regulatory loop.

The described data indicate EGR1 and RXRA as the key link between the TGF-β-induced pathways and CCL2 expression and allow us to propose a positive regulatory loop (Fig. 7). Research previously described that high CCL2 levels directly correlated with intense macrophage polarization to the M2 phenotype and secretion of CCL22, which attracts and accumulates Th2 cells^14^. The presence of M2 macrophages is associated with the general anti-inflammatory response^33^ and with production of TGF-β, which could cause further stimulation of CCL2 expression in breast cancer cells via EGR1 and RXRA. The participation of the EGR1 and RXRA transcription factors in such positive regulatory loops is in agreement with their anti-inflammatory functions. EGR1 is known to activate expression of a range of anti-inflammatory genes, including IL4^34,35^, and is also involved in the positive regulation of TGF-β transcription^36^. In its turn, RXRA provides an anti-inflammatory effect by repressing transcription of IL-12 and TNF-α and induction of IL-10^37^. Therefore, activation of CCL2 expression could be an anti-inflammatory effect of these factors that was not previously described.

Special attention should be paid to the fact that both RXRA and EGR1 are independently essential for TGF-β-dependent activation of the CCL2 gene promoter and subsequent induction of CCL2 expression. We did not set out to reveal the precise mechanism of participative action of these factors; however, we could propose a possible model (Fig. 7) based on the existing information and on our CHIP and gene expression data (Fig. 5). EGR1 is known to be a rapid-acting transcription factor that directly binds to the activated SMAD3, translocates to the nucleus, and performs transcriptional activation of the target genes^30^. The specific involvement of SMAD3 via interaction with EGR1 may explain why SMAD3 knockdown specifically abrogates the TGF-β-induced activation of CCL2 promoter (Fig. 2E). Accordingly, we did not observe induction in EGR1 expression by TGF-β (Fig. 4A,C), but registered rapid changes in its binding to CCL2 promoter (Fig. 5). In addition, EGR1 is reported to act as an epigenetic modulator in different tissues^38,39^, including breast cancer^40^, which results in the ability to influence the methylation context around its binding region and significantly change the affinity of other factors to this region^40^.

In addition to previously described anti-inflammatory functions of EGR1, its action could also provide some features of the pro-inflammatory response, including NF-kB activation^41^, and is inhibited by the secondary long-lasting processes triggered by TGF-β-dependent signaling. For example, TGF-β induces LXRA, RXRA, and RARA expression levels^42^. These factors are known to produce the overall anti-inflammatory response^37,43^ and, particularly, reduce EGR1 expression levels^44^. These processes presumably lead to a decrease in EGR1 levels and to subsequent involvement of RXRA in the activation of the CCL2 promoter. Indeed, RXRA binding activity correlates well with its expression level (Fig. 5). In summary, rapid EGR1 action in response to TGF-β could lead to both activation of the CCL2 promoter and modification of its epigenetic context but also is inhibited itself in a short time. RXRA, on the contrary, could bind to the prepared CCL2 promoter region and provide long-lasting activation effects. Thereby, both EGR1 and RXRA could be necessary for complex activation of the CCL2 promoter in response to TGF-β.

We conclude that, in this study, we provided a possible mechanism that underlies TGF-β-dependent regulation of the CCL2 gene promoter in triple-negative advanced breast cancer cells.

## Materials and methods

### Cell lines

MDA-MB-231 and HCC1937 cell lines were maintained in a local stock of the Shemyakin–Ovchinnikov Institute of Bioorganic Chemistry RAS (Moscow, Russia) and kindly provided by Dr. D.S. Kravchenko. The stocks of MDA-MB-231 and HCC1937 cells were recently authenticated using commercially performed STR analysis (Gordiz, Moscow, Russia, data not shown). Both cell lines were cultured in Dulbecco’s modified Eagle’s medium (DMEM, Paneco, Moscow, Russia) supplemented with 10% fetal bovine serum.

### Ethical approval

Scientific Council of the Engelhardt Institute of Molecular Biology declared no ethical approval requirements for experiments performed on the commercially available cell lines used in the study.

### RNA isolation, reverse transcription, and real-time quantitative RT-PCR

The total RNA was extracted from cells using ExtractRNA reagent (Evrogen, Moscow, Russia) according to the manufacturer’s protocol. cDNA was generated using the M-MULV reverse transcriptase and oligo-dT primer from the MMLV RT kit (Evrogen, Moscow, Russia). Quantitative RT-PCR was performed using the protocol and PCR program described earlier^45^ and specific primers designed to amplify intron-spanning fragments of human β-actin, CCL2, CXCR4, IL11, EGR1, EGR2, PAX6, RARA, RXRA, and SP1 genes (Supplementary Table S1). The results were calculated using ΔCt method, and all expression levels of target genes were normalized to β-actin.

### Western blot analysis

The protocols for the total cell lysate preparation, electrophoresis, transfer to the nitrocellulose membrane, and band visualization were described earlier^15^. For analysis of EGR1 and RXRA protein levels we used nuclear extracts prepared according to the previously described protocol in the same manner as for the pull-down assay^32^. The membranes were pre-blocked using 3% BSA and incubated with anti-pSMAD3 (Ser204) antibodies (PA5-36877, Thermo Fisher Scientific, Waltham, MA, USA) at a 1:2000 dilution, anti-CCL2 antibodies (ab9669, Abcam, Cambridge, UK) at a 1:2000 dilution, anti-pSMAD2 (Ser465/467) antibodies (138D4, Cell Signaling Technology, Danvers, MA, USA) at a 1:2000 dilution, anti-SMAD4 antibodies (D3R4N, Cell Signaling Technology, Danvers, MA, USA) at a 1:3000 dilution, anti-SMAD6 antibodies (sc-25321, Santa Cruz Biotechnology, Santa Cruz, CA, USA) at a 1:2000 dilution, anti-SMAD7 antibodies (ab216428, Abcam, Cambridge, UK) at a 1:3000 dilution, anti-EGR1 antibodies (44d5, Cell Signaling Technology, Danvers, MA, USA) at a 1:3000 dilution, anti-RXRA antibodies (d6h10, Cell Signaling Technology, Danvers, MA, USA) at a 1:3000 dilution, anti-PCNA antibodies (D3H8P, Cell Signaling Technology, Danvers, MA, USA) at a 1:3000 dilution as a loading control for nuclear extracts and anti-β-actin antibodies (ab8229, Abcam, Cambridge, UK) at a 1:3000 dilution as a loading control for whole-cell lysates. Gel images of three independent repeats were analyzed using ImageJ software to determine integral densities of the bands. The values of integral densities to β-actin for CCL2 and pSMAD3 were normalized and to PCNA for RXRA and EGR1.

### Molecular cloning and luciferase reporter constructs

We amplified a potential full-size CCL2 promoter region (Prom 1) located within positions − 1196 and + 488 in response to the CCL2 transcription start site (TSS) by PCR using human genomic DNA isolated from MDA-MB-231 cells and specific primers containing HindIII и NcoI restriction sites (Supplementary Table S1). The Prom 1 region was cloned to a pGL3 base luciferase reporter vector (Promega, Madison, WI, USA). We assembled luciferase reporter plasmids containing deletion variants of CCL2 promoter: Prom 2 (− 735/+ 488), Prom 3 (− 563/+ 488), Prom 4 (− 443/+ 488), Prom 5 (− 244/+ 488), and Prom 6 (− 1/+ 488), (Schematically illustrated on the Fig. 2); and a Prom 1 variant with point mutations in the predicted EGR1 and RXRA binding sites (Schematically illustrated in Fig. 6A).

### Luciferase reporter assay

MDA-MB-231 and HCC1937 cells were electroporated with 10 μg of purified plasmid DNA and 0.1 μg of pRL-CMV Renilla luciferase control reporter vector (Promega, Madison, WI, USA) using the Neon Transfection System (Thermo Fisher Scientific, Waltham, MA, USA) and the following regimens: four 10 ms 1400 V pulses for MDA-MB-231, and three 10 ms 1550 V pulses for HCC1937. Firefly luciferase activity was measured as described earlier^46^, and normalized to the activity of Renilla luciferase in order to account for the variations in cell transfection efficiency.

### Knockdown of studied transcription factors and the proteins of SMAD family using siRNA

We used previously published siRNAs against SMAD2, SMAD3^47^ and SMAD4^48^ proteins and EGR1, EGR2, PAX6, RARA, RXRA, and SP1 transcription factors^49^. The sequences of the siRNAs are represented in Supplementary Table S1. Commercially synthesized single-stranded RNAs (Syntol, Moscow, Russia) were annealed as previously described^50^. MDA-MB-231 and HCC1937 cells were transfected with siRNA duplexes (500 pmol of per 5 million cells) 48 h before transfection with the luciferase constructs and a further 200 pmol to extend the silencing effect.

### Chromatin immunoprecipitation (ChIP) assay

We applied the cross-linking chromatin immunoprecipitation (X-ChIP) protocol as described earlier^51^. Briefly, after the lysis of 2 × 10^7^ of MDA-MB-231 or HCC1937 cells containing protein-DNA complexes preliminary cross-linked with 0.75% formaldehyde, these complexes were sonicated to obtain average DNA fragment sizes of 700 bp, incubated with antibodies to RXRA (d6h10, Cell Signaling Technology, Danvers, MA, USA) or EGR1 (44d5, Cell Signaling Technology, Danvers, MA, USA), and precipitated with pre-blocked protein A sepharose beads. After the elution of protein-DNA complexes and DNA purification, the target DNA was quantified by real-time PCR. We used four types of controls: a background control without lysate; a nonspecific precipitation control without antibodies; a control with isotype rabbit IgG antibodies (DA1E, Cell Signaling Technology, Danvers, MA, USA); and an amplicon from a nonspecific locus containing no predicted RXRA- or EGR1-binding sites. The primer sequences are represented in Supplementary Table S1. None of the control precipitations produced signals above the background level.

### Pull-down assay

We performed a pull-down assay according to our previously published original protocol^32^. Briefly, we amplified a 245-bp fragment of CCL2 promoter (− 244/− 1) and the same fragment with point mutations in EGR1/RXRA binding sites (Fig. 6B). An amplicon from a non-specific locus of the same length that did not contain any predicted EGR1/RXRA binding sites served as a negative control. The primer sequences are represented in Supplementary Table S1. The protocols of the isolation of nuclear extracts from MDA-MB-231 and HCC1937 cell lines, the immunoprecipitation of protein-DNA complexes, and quantification of bound DNA probes by real-time PCR were described earlier^32^. For precipitation, we used anti-RXRA (d6h10, Cell Signaling Technology, Danvers, MA, USA) and anti-EGR1 (44d5, Cell Signaling Technology, Danvers, MA, USA) antibodies. We used the following controls to calculate the background signal level: with an amplicon from a nonspecific locus; without nuclear extract; without antibodies; and isotype control with rabbit IgG (DA1E, Cell Signaling Technology, Danvers, MA, USA).

### Statistical analysis

We used Microsoft Excel and Graphpad Prism software for our statistical analyses. Statistical significance was determined using a two-tailed unpaired Student’s t-test. The data are represented as the mean ± SD.

## Supplementary Information

Supplementary Information.

## Abbreviations

ACTB: Beta-actin
CCL2: C–C motif ligand 2
cDNA: Complementary DNA
CXCR4: C-X-C chemokine receptor type 4
EGR1: Early growth response protein 1
EGR2: Early growth response protein 2
IgG: Immunoglobulin G
IL11: Interleukin 11
MMLV RT: Moloney murine leukemia virus reverse transcriptase
M-MULV: Moloney murine leukemia virus
PAX6: Paired box protein 6
PCNA: Proliferating cell nuclear antigen
RARA: Retinoic acid receptor alpha
RXRA: Retinoid X receptor alpha
SEM: Standard error of the mean
siRNA: Small interfering RNA
SMAD3: Mothers against decapentaplegic homolog 3
SP1: Specificity protein 1
STR analysis: Short tandem repeat analysis
