# Supplementary material for: EGR1 and RXRA transcription factors link TGF-β pathway and CCL2 expression in triple negative breast cancer cells
Source: Sci Rep. 2021 Jul 8;11:14120. doi: 10.1038/s41598-021-93561-6 (PMC8266896; doi:10.1038/s41598-021-93561-6)
Supplement: Supplementary file 1 — Supplementary Information. [file 41598_2021_93561_MOESM1_ESM.docx]

**Supplementary material for:**

**EGR1 and RXRA transcription factors link TGF-β pathway and CCL2 expression in triple negative breast cancer cells**

Alisa M. Gorbacheva^1^, Aksinya N. Uvarova^1^, Alina S. Ustiugova^1^, Arindam Bhattacharyya^2^, Kirill V. Korneev^1^, Dmitry V. Kuprash^1,3^, Nikita A. Mitkin^1,*^

^1^ Center for Precision Genome Editing and Genetic Technologies for Biomedicine, Engelhardt Institute of Molecular Biology, Russian Academy of Sciences

^2^ Immunology Laboratory, Department of Zoology, University of Calcutta, 35, Ballygunge Circular Road, Kolkata, 700019, West Bengal, India

^3^ Biological Faculty, Lomonosov Moscow State University, Moscow 119234, Russia

^*^ Correspondence: mitkin.n.a@gmail.com (N.A.M); Tel.: +7-910-483-99-32

Table of Contents: page

Supplementary Table 1 2

Supplementary Figure 2 3

Supplementary Figure 1 4

Supplementary Figure 2 5

**Supplementary table 1.**

| Primers for amplification of CCL2 promoter variants and point mutagenesis in CCL2 promoter | |
| --- | --- |
| Prom1 fw | TATAAGCTTTTCTGAGGAAATCTAAGGCA |
| Prom2 fw | TATAAGCTTGGAATTTACAGAGTTAATAT |
| Prom3 fw | TATAAGCTTAGTTCTGCTAGGCTTCTATG |
| Prom4 fw | TATAAGCTTATGTGGCCTGAAGGTAAGCT |
| Prom5 fw | TATAAGCTTAAAGTGTCTCGTCCTGACCC |
| Prom6 fw | TATAAGCTTAACTGAAGCTCGCACTCTCGCC |
| Prom rv | TATCCATGGTCATTACCTCTGCAGAGTCA |
| Mut fw | CTAAAGTCACTTTTCTGCCCGCTTTCAATA |
| Mut rv | AAAGTGACTTTAGGAGTCAAGCAGGAGGAG |
|  |  |
| Primers for amplification of fragments for pull-down and ChIP assays | |
| Pull-ChIP fw | AAAGTGTCTCGTCCTGACCC |
| Pull-ChIP rv | CGAGAGTGCGAGCTTCAGTT |
| Pull-ChIP contr fw | TGATCTCGAACTCCTGACCT |
| Pull-ChIP contr rv | GTGCTGTGATTACAGGTGTG |
|  |  |
| Primers for real-time RT-PCR | |
| CCL2 fw | CATTGTGGCCAAGGAGATCTG |
| CCL2 rv | CTTCGGAGTTTGGGTTTGCTT |
| CXCR4 fw | GGCAGAGGAGTTAGCCAAGAT |
| CXCR4 rv | TAGTGGGCTAAGGGCACAAG |
| IL11 fw | TGAGCCTGTGGCCAGATACA |
| IL11 rv | CTGGGAATTTGTCCCTCAGC |
| EGR1 fw | GGATCCTTTCCTCACTCGCC |
| EGR1 rv | TCAGGAAAAGACTCTGCGGTC |
| EGR2 fw | TGGTGTGTGTGGTGGTTGTT |
| EGR2 rv | TGTTGATCATGCCATCTCCG |
| PAX6 fw | GAGAGCGAGCGGTGCATTTG |
| PAX6 rv | AGAGCAATTCTCAGATTCCTATGCT |
| RARA fw | CACACACCTGAGCAGCATCAC |
| RARA rv | GATGTCTCAAGAGCCGGTCC |
| RXRA fw | TTTCCTGCCGCTCGATTTCT |
| RXRA rv | ACCTTGAGGACGCCATTGAG |
| SP1 fw | CCACCATGAGCGACCAAGAT |
| SP1 rv | GTAGCCCCAGAGGAGGAAGA |
| β-actin fw | TGCGTGACATTAAGGAGAAG |
| β-actin rev | GTCAGGCAGCTCGTAGCTCT |
|  |  |
| siRNAs for genes expression knockdown | |
| EGR1Sense | CCAUGGACAACUACCCUAAtt |
| EGR1Anti | UUAGGGUAGUUGUCCAUGGtg |
| EGR2Sense | GAUCCACCUGAGACAGAAAtt |
| EGR2Anti | UUUCUGUCUCAGGUGGAUCtt |
| PAX6sense | CCAACUCCAUCAGUUCCAAtt |
| PAX6anti | UUGGAACUGAUGGAGUUGGta |
| RARAsense | GAAGAUUACUGACCUGCGAtt |
| RARAanti | UCGCAGGUCAGUAAUCUUCat |
| RXRAsense | AGGACUGCCUGAUUGACAAtt |
| RXRAanti | UUGUCAAUCAGGCAGUCCUtg |
| SP1sense | GCAACAUGGGAAUUAUGAAtt |
| SP1anti | UUCAUAAUUCCCAUGUUGCtg |
| SMAD2sense | GUCCCAUGAAAAGACUUAAtt |
| SMAD2anti | UUAAGUCUUUUCAUGGGACtt |
| SMAD3sense | GGAGAAAUGGUGCGAGAAGtt |
| SMAD3anti | CUUCUCGCACCAUUUCUCCtc |
| SMAD4sense | GAUGAAUUGGAUUCUUUAAtt |
| SMAD4anti | UUAAAGAAUCCAAUUCAUCtt |

**Supplementary table 2.**

mRNA expression data (Affymetrix) from the CCLE database. The values (RMA, log2) above 4 should be considered as significant expression (Barretina et al., 2012).

|  | MDA-MB-231 | HCC1937 |
| --- | --- | --- |
| EGR1 | 7.109442 | 7.920931 |
| EGR2 | 4.804946 | 4.445929 |
| PAX6 | 4.196315 | 8.602077 |
| RXRA | 7.151621 | 7.819896 |
| RARA | 4.999526 | 4.876988 |
| SP1 | 6.314904 | 6.831742 |

Barretina, J., Caponigro, G., Stransky, N., Venkatesan, K., Margolin, A. A., Kim, S., ... & Reddy, A. (2012). The Cancer Cell Line Encyclopedia enables predictive modelling of anticancer drug sensitivity. *Nature*, *483*(7391), 603-607.

**Figure S1.** Full-size images of western blots of CCL2, β-actin, Phospho-SMAD3 (Ser204), Phospho-SMAD2 (Ser465/467), SMAD4, SMAD6 and SMAD7 in MDA-MB-231 cells

**
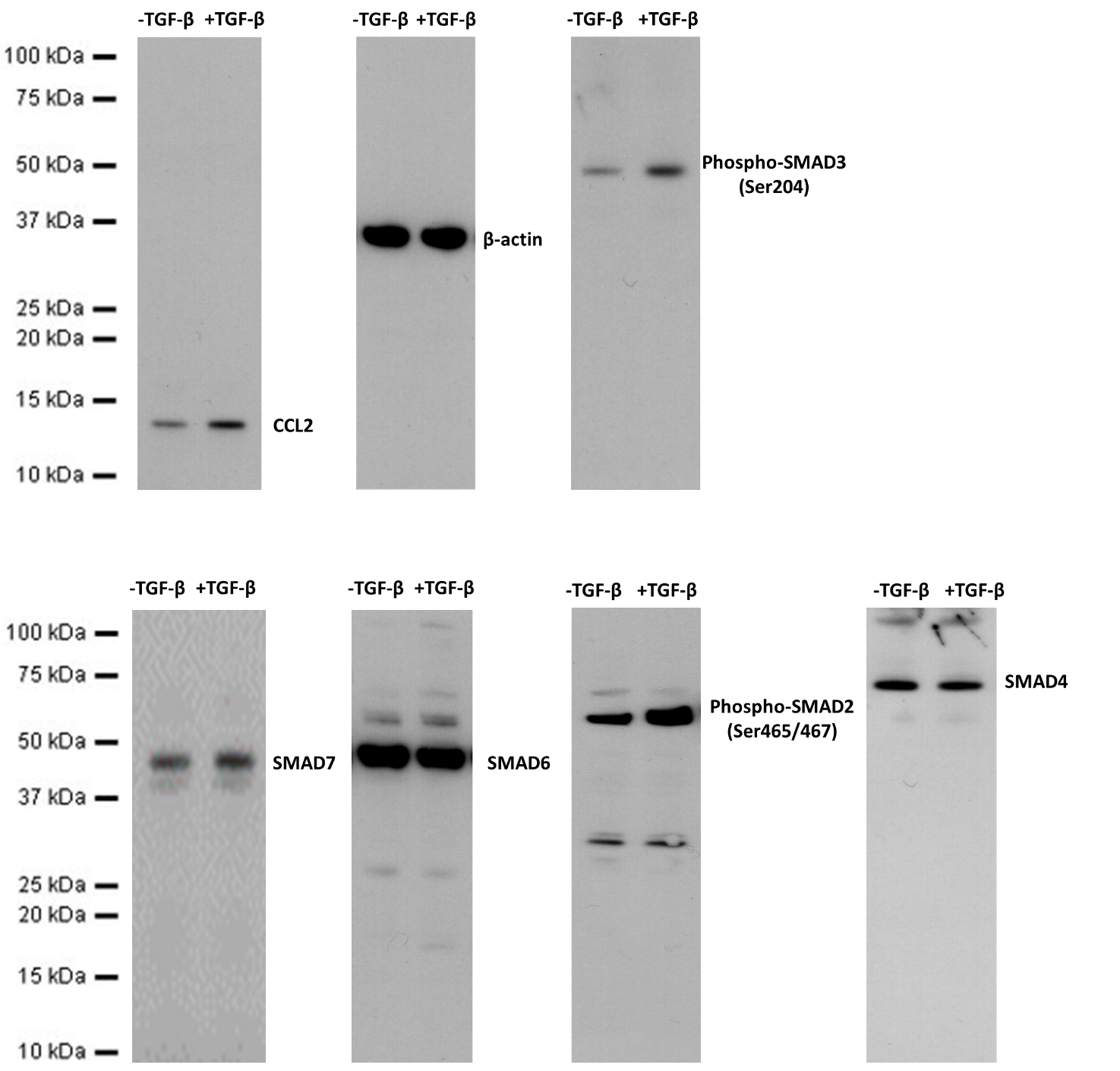
**

The images of the same membrane stained with anti-CCL2, anti-β-actin and anti-Phospho-SMAD3 (Ser204) respectively are represented. For phospho-SMAD2 (Ser465/467), SMAD4, SMAD6 and SMAD7 we used the same lysates as in case of CCL2, β-actin and Phospho-SMAD3 (Ser204).

**Figure S2.** Full-size images of western blots of RXRA and EGR1 with respective PCNA bands in MDA-MB-231 cells


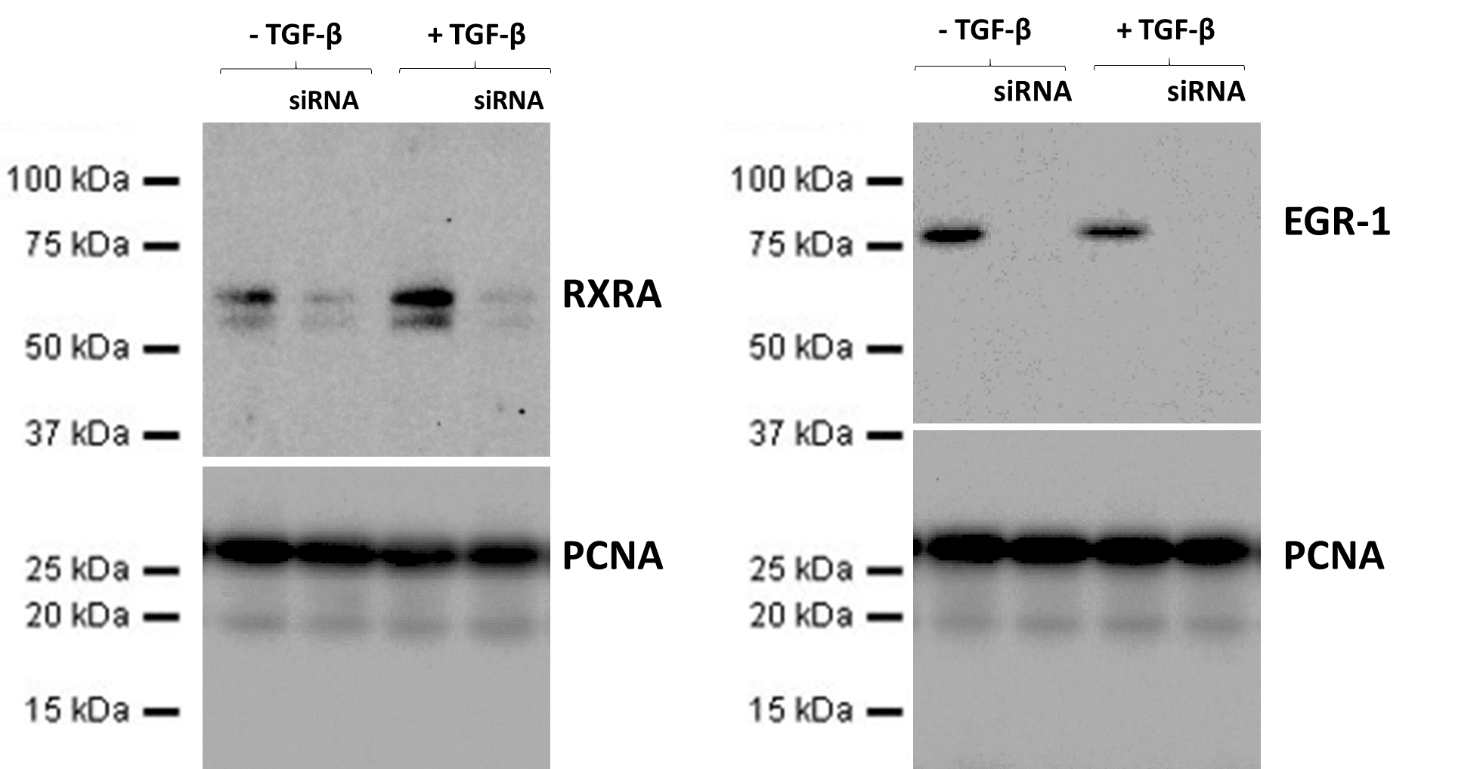


Each membrane was splitted horizontally into two pieces, then the upper piece was stained with RXRA/EGR1 antibodies and the bottom piece was stained with PCNA antibodies.
